# Honest and True New York Doctors

**Published:** 1883-04

**Authors:** 


Honest and True New York Doctors.—An association for the purpose of upholding the old code of medical ethics, and resisting any modification of this code that does not emanate from the body in which it originated, has been formed, and held its first regular meeting at the hall of the Academy of Medicine on Friday evening, March 23d. Among those interested in the movement are such leading men as Austin Flint, sr., Austin Flint, jr., T. Gaillard Thomas, Alonzo Clark, Willard Parker, Thomas M. Markoe, Lewis A. Sayre, Frank H. Hamilton, Isaac E. Taylor, William T. Lusk, Samuel S. Purple, Abram Du Bois, J. W. S. Gouley, John H. Hinton, Stephen Smith, J. Lewis Smith, Jared Linsey, Nathan Bozeman, Henry D. Noyes, Richard H. Derby, F. D. Weisse, J. Williston Wright, Octavius A. White, John G. Adams, and William J. Morton.—*Boston Medical and Surgical Journal.*

